# Indicators of induced subacute ruminal acidosis (SARA) in Danish Holstein cows

**DOI:** 10.1186/s13028-015-0128-9

**Published:** 2015-07-17

**Authors:** Anne Mette Danscher, Shucong Li, Pia H Andersen, Ehsan Khafipour, Niels B Kristensen, Jan C Plaizier

**Affiliations:** Department of Large Animal Sciences, Faculty of Health and Medical Sciences, University of Copenhagen, Højbakkegård Alle 5, 2630 Taastrup, Denmark; Department of Animal Science, Faculty of Agricultural and Food Sciences, University of Manitoba, 201-12 Dafoe Road, Winnipeg, MB R3T 2N2 Canada; Department of Clinical Sciences, Faculty of Veterinary Medicine and Animal Science, Swedish University of Agricultural Sciences, Ulls väg 14C (Ultuna), 756 51 Uppsala, Sweden; Department of Medical Microbiology, Faculty of Health Sciences, University of Manitoba, 745 Bannatyne Avenue, Winnipeg, MB R3E 0J9 Canada; Knowledge Centre for Agriculture, Cattle, SEGES, Agro Food Park 15, 8200 Aarhus N, Denmark

**Keywords:** Subacute ruminal acidosis, SARA, Bovine, Biomarkers, Diagnosis

## Abstract

**Background:**

The prevalence of subacute ruminal acidosis (SARA) in dairy cows is high with large impact on economy and welfare. Its current field diagnosis is based on point ruminal pH measurements by oral probe or rumenocentesis. These techniques are invasive and inaccurate, and better markers for the diagnosis of SARA are needed. The goal of this study was to evaluate clinical signs of SARA and to investigate the use of blood, faecal and urinary parameters as indicators of SARA. Six lactating, rumen cannulated, Danish Holstein cows were used in a cross-over study with three periods. The first and second periods included two cows on control diet and two cows on nutritional SARA challenge. The third period only included two cows on SARA challenge. Control diet was a conventional total mixed ration [45.5% dry matter (DM), 17.8% crude protein, 43.8% neutral detergent fibre, and 22.5% acid detergent fibre (DM basis)]. SARA challenge was conducted by substituting control diet with grain pellets (50% wheat/barley) over 3 days to reach 40% grain in the diet. Ruminal pH was measured continuously. Blood samples were collected once daily at 7 h after feeding. Samples of faeces and urine were collected at feeding, and at 7 and 12 h after feeding. Blood samples were analysed for pCO2, pO2, pH, electrolytes, lactate, glucose, packed cell volume (PCV), and total plasma protein concentration. Milk composition, ruminal VFA, and pH of faeces and urine were measured.

**Results:**

SARA was associated with decreased (*P* < 0.05) minimum ruminal, faecal and urinary pH. Daily times and areas of ruminal pH below 5.8, and 5.6 were increased to levels representative for SARA. Significant differences were detected in milk composition and ruminal VFAs. Blood calcium concentration was decreased (*P* < 0.05), and pCO_2_ tended to be increased (*P* = 0.10). Significant differences were not detected in other parameters.

**Conclusions:**

SARA challenge was associated with changes in faecal and urinary pH, blood calcium concentration and pCO_2_. These may be helpful as indicators of SARA. However changes were small, and diurnal variations were present. None of these parameters are able to stand alone as indicators of SARA.

## Background

Subacute ruminal acidosis (SARA) has been defined as impaired ruminal health, during which a reversible ruminal pH depression occurs [1–4]. Using rumenocentesis and a ruminal pH threshold of 5.5, the prevalence of SARA in intensive dairy production has been found to range between 11 and 26% [1–4] up to over 40% in some herds [1]. The definition and ruminal pH threshold for SARA vary among studies, but it is generally agreed that SARA occurs when the ruminal pH is lower than 5.5–5.8 for several hours a day [4]. Ruminal fluid pH decreases because the ruminal microbes convert carbohydrates to short chain fatty acids at a rate that exceeds the rumen’s absorptive, buffering and outflow capacity [4]. This leads to a changes in the ruminal microbial populations, reduced fibre digestion [5], and has been related to decreased feed intake [4, 6] and milk fat production [4, 7], alterations in the biohydrogenation of unsaturated fat in the rumen and the profile of unsaturated and odd and branch chain fatty acids in the milk fat [3, 8], systemic inflammation and localized inflammation of the tissues of the papillae of the rumen [4], liver abscesses [9, 10], and SARA has been suspected to lead to claw horn lesions and lameness [11, 12]. Economic losses due to reduced production alone have been estimated to 400 US $ per cow per lactation [4]. Thus, SARA is not a well-defined diagnosis, but rather a syndrome, or a set of signs, associated with low ruminal pH and poor ruminal health. In the following “SARA” refers to this syndrome or set of SARA related signs.

Many cases of SARA may not be detected, as the current field diagnosis of SARA is not clearly defined and depend either on point ruminal pH measurements, which are invasive and due to fluctuations in pH not very accurate or sensitive for the diagnosis of a longer lasting pH depression indicative of SARA, or on continuous measurements which require costly equipment, and are primarily suited for research purposes [4]. Additionally, some studies suggest that a ruminal pH depression alone is not enough to result in the clinical signs related to SARA [13]. Hence the use of the ruminal pH as the sole indicator of SARA related signs and ruminal health should be avoided. Easily accessible and inexpensive markers of SARA are therefore needed for the diagnosis of ruminal health problems. Various analyses of blood, urine, faeces, and milk have been considered and evaluated for this purpose [4, 14, 15], but the results of these studies are conflicting.

Consequently, the purpose of this study was to evaluate clinical, including orthopaedic, signs of SARA and to determine if analyses of blood, ruminal fluid, faeces, and urine might be used as indicators of SARA. This work is a part of a larger study during which various measurements on biological fluids and solids, including microbiological and proteomic analyses, are evaluated for the diagnosis of impaired ruminal health.

## Methods

### Animals, feeding and experimental setup

Six primiparous, rumen cannulated, Danish Holstein cows between 200 and 300 days in milk were used in this study. Jugular catheters (EQUIVET HiFlow LongTerm IV Catheter 14G × 5.25″, Jørgen Kruuse A/S, Langeskov, Denmark) were placed in the left jugular veins of the cows, and were flushed with heparinised saline after each blood collection. The cows were housed in individual tie stalls on concrete floors with rubber mats and bedded with wood shavings and had ad libitum access to drinking water. Feed was offered ad libitum twice a day at 8.00 and 14.30 h aiming at 5–10% orts.

The cows received a conventional total mixed ration (TMR) and had been adapted to that diet for at least 6 weeks prior to the trial (Table 1). SARA challenges were conducted by substituting TMR with grain pellets (50% wheat/barley (WB), ground and pelleted) gradually over 3 days to reach 40% grain of total ration dry matter (Table 2).

**Table 1:** Composition of total mixed ration used in the study

Ingredients	% as fed	% of DM
Barley straw	0.6	1.2
Rapeseed cake	7.3	14.6
Vitamin/mineral mix (“Komix”)	0.7	1.4
Urea	0.3	0.6
Beet pellets	2.8	5.4
Water	4.0	0.0
Grass silage	22.3	24.1
Corn silage	60.3	49.3
Soy bean meal	1.8	3.6

**Table 2:** Diet ingredients and chemical composition

	Control diet	SARA diet
Ingredients (% of DM)		
TMR	100	60
Wheat/barley pellets	0	40
Forage	75	45
Chemical composition (% of DM)		
DM %	45.1	55.6
Crude protein	17.8	15.0
Crude fat	3.5	4.6
NDF	43.8	31.3
Forage NDF	26.5	15.9
ADF	22.4	16.2
Starch	19.6	31.8
Ash	8.8	6.1
Ca (g/kg)	6.6	4.2
P (g/kg)	3.8	3.5
K (g/kg)	15.1	10.9
Mg (g/kg)	2.4	1.9
Na (g/kg)	2.2	1.4
DCAD (mEq/kgDM)	272.1	213.3

Ingredients and mean chemical composition of diets fed to control cows and before challenge (control), and during SARA challenge (SARA).

The trial timeline included 3 control days (TMR diet), 3 transition days (TMR + increasing percentages of WB pellets), 4 SARA days (40% WB pellets), and 6 recovery days (TMR). Control cows received TMR diet during the entire period (Table 3). The trial was designed as a cross over study and was carried out in three blocks (Table 4). Two blocks included two cows on control diet and two on SARA diet in each block. A third block included two cows on SARA diet. Cows that had previously been subjected to the SARA treatment (cows 2 and 4) were given a 6-week “wash-out” before they entered the trial again (Table 4).

**Table 3:** Trial timeline

Day of trial	1	2	3	4	5	6	7	8	9	10	11	12	13	14	15	16
Period	Control			Transition			SARA				Recovery					
Group																
SARA	TMR diet			WB Step up			TMR + WB pellets				TMR diet					
Control	TMR diet			TMR diet			TMR diet				TMR diet					

The trial included 3 control days (TMR diet), 3 transition days (TMR + increasing percentages of grain (WB) pellets), 4 SARA challenge days (40% grain pellets), and 6 recovery days (TMR). Control cows received TMR diet during the entire period.

**Table 4:** Block structure and ID of the six Danish Holstein cows included in the study

Cow ID	Block	1				2				3		
	Week of year	18	19	20	21	22	23	24	25	26	27	28
2		Control	SARA							Control	SARA	
4		Control	SARA							Control	SARA	
1		Control	Control			Control	SARA					
3		Control	Control			Control	SARA					
5						Control	Control					
6						Control	Control					

The objective was to create a reversible ruminal pH depression below 5.6 for more than 180 min/day, preferably without reaching ruminal pH values below 5.2 [4, 6].

### Feed

The amounts of feed offered and refused were recorded daily. Samples of the offered TMR were collected daily and stored at −20°C and pooled per block. NDF (neutral detergent fibre), ADF (acid detergent fibre), ADL (acid detergent lignin), were analysed at the laboratory at Department of Large Animals Sciences, University of Copenhagen using an Ankom220 fiber analyzer, Ankom Technology, Macedon, NY, USA [16], including alpha amylase for NDF analysis. Other feed analysis was carried out at a commercial laboratory (Eurofins-Steins, Holstebro, Denmark) [Crude protein: AOAC 1992, method 992.23, Starch (YSI Incorporated Life Sciences, Ohio, USA), Ash: 2009/152/EF, Ca, P, K, Mg, Na: DS13805:2002-ICP-OES]. Samples of orts from each cow were collected daily, stored at −20°C and pooled by control and SARA challenge periods for each animal. DM content was determined by drying in a forced air oven at 60°C for 48 h. Daily dry matter intake (DMI) was determined by subtracting DM refused from DM offered.

### Clinical examination

Clinical examinations were performed midmornings and included evaluation of general demeanour (1: bright, alert and responsive, 2: slightly depressed, 3: depressed, 4: very depressed, 5: moribund), auscultation of ruminal contractions (subjective evaluation, with four points. 4: good, 3: decreased, 2: very decreased, 1: absent), faeces consistency (1: runny liquid, splatters spreads readily, 2: loose may pile slightly, splatters moderately, 3: soft firm, piles but spreads slightly, 4: dry hard, not distorted on impact [17]), and measuring rectal temperature, heart rate, and respiration rate.

### Orthopaedic examination

The cows were handled and trained to allow lameness examination while being led by hand, as well as testing for presence of pain in the claws of the front limbs. Due to safety issues, claw testing was not performed on the hind limbs.

Weight-shifting was defined as the shifting of weight laterally from one limb to another in a monotonous repeated manner, without any obvious other external cause. Weight shifting was scored as observed or not observed.

Coronary band temperature was measured after clipping an area of 3 × 3 cm of the skin just proximal to the coronary band on the dorsolateral aspect of the lateral claw on one hind limb and on the proximal part of the right thigh. Skin temperatures of the coronary band (CBT) and thigh (ST) were measured using an infrared thermometer (model 576, Fluke Corporation, Washington, USA) with an emission coefficient of 0.98. The thermometer to skin distance was kept constant at 30 cm; the skin was wiped or brushed and left to dry if dirty or wet, and cows lying down were encouraged to stand and temperatures measured after at least ten minutes standing. Rectal and room temperatures were also measured at this time.

Claw testing was performed over the typical site of sole ulcer (axial sole-bulb junction) and the central part of the dorso-abaxial claw wall of the front limbs by applying a large standard hoof-tester constructed with a force measuring device (KERN FK1K, Kern Kraft & Sohn, Balingen, Germany) welded on to one of the arms. Just enough pressure was applied to visually appreciate the sole horn yield. An aversive reaction was defined as an attempt to withdraw the leg when force was applied, and two consecutive aversive reactions were classified as positive. Hoof testing was performed and coronary band temperature measured on days 1, 3, 6, 8, 10, 11, and 15 (Table 5).

**Table 5:** Structure for sampling and examination during each block of the study

Day of trial	Time (h)	1	2	3	4	5	6	7	8	9	10	11	12	13	14	15	16
		Control			Transition			SARA				Recovery					
Ruminal fluid, urine, feces	9.00, 15.00, 21.00	×		×				×	×		×						
	15.00		×				×			×			×		×		×
Blood	15.00	×		×				×	×	×	×				×		
Synovia, rumen papillae												×					
Clinical examination		×	×	×			×	×	×	×	×	×	×		×		×
Hoof testing, coronary band temperature		×		×			×		×		×	×				×	
Locomotion score		×		×			×			×	×					×	

Locomotion assessment was performed while cows were led in a walk on a hard, slightly uneven surface by one or two observers on days 1, 3, 6, 9, 10, and 15 (Table 5) according to Sprecher et al. [18] (1: Normal, 2: Mildly lame, 3: Moderately lame, 4: Lame, 5: Severely lame.).

### Ruminal pH

Ventral sack ruminal pH was measured continuously by indwelling pH meter probes that allowed for wireless downloading of data (eCow Rumen Analyzer, Exeter, United Kingdom). The probes were attached to 1 kg rounded stainless steel weights in order to keep them positioned in the ventral sack and their position was checked daily. The pH was measured every minute, and averaged over 5 min before storage. Daily mean, minimum and maximum ruminal pH as well as time and area under the curve below pH 6.0, 5.8, 5.6, and 5.2 during the SARA period were calculated as described by Gozho et al. [6].

### Rumen, urine and faecal samples

Samples of ruminal fluid, faeces and urine were collected at 9.00, 15.00 and 21.00 h on control days 1 and 3 and on SARA challenge days 7, 8, and 10 and at 15.00 h at days 2, 6, 9, 12, 14, and 16 (Table 5).

Ruminal fluid samples were collected by introducing a closed 100 ml plastic bottle through the cannula into the ventral ruminal sack, opening it and allowing it to fill up, before retrieving it. Samples were filtered through two layers of cheesecloth and 4 ml were immediately transferred to a cryo vial containing 1 ml 25% meta phosphoric acid, mixed and stored at −20°C, until analysed for VFAs and l-lactate by gas chromatography [19].

Faecal samples were obtained directly from the rectum. Urination was stimulated by perineal stimulation and samples were obtained. In all samples, pH was measured immediately after collection (Cardy Twin pH Meter, Spectrum Technologies Inc., Plainfield, IL, USA). A 2-point calibration (pH 4 and 7) was performed before the measurements.

### Blood samples

Blood samples were collected once daily at 15.00 h on control days 1 and 3, on SARA days 7, 8, 9, and 10, and on recovery day 14 (Table 5). Maintaining aseptic procedures, blood samples were collected from the jugular catheters once daily. Ten ml of blood was extracted and discarded before sampling 60 ml and finally the catheter was flushed with 8 ml heparinized (100 IU/ml) saline. Blood samples were analysed within 30 min for pCO2, pO2, pH, Na^+^, K^+,^ Ca^++^, Cl^−^, L-lactate, and glucose concentrations in a stationary blood gas analysing apparatus (ABL 725, Radiometer, Brønshøj, Denmark) using heparin-stabilized blood. Packed cell volume (PCV) was measured on heparin-stabilized blood using Bets Micro Haematocrit Tubes (Vitrex Medical A/S, Herlev, Denmark, and Micro 20, Hettich Zentrifugen, Bie & Berntsen A/S, Rødovre, Denmark).

### Milk yield and samples

Cows were milked twice a day at 8.00 and 14.30 h using a transportable milking machine with attached bucket. Daily milk yields were measured by weight. Samples for measurements of milk fat, protein, and somatic cell count were taken from the bucket after thorough mixing of the milk at milking time on two control and two SARA days in block 2 and 3. Analysis was performed at a commercial laboratory (Eurofins-Steins, Holstebro, Denmark) (milk fat and protein: infrared spectroscopy, somatic cell count: flow cytometry, CombiFoss, Foss, Hillerød Denmark).

### Data analysis and statistical methods

The overall hypothesis was that there is a difference in the parameters measured between the cows that received the SARA challenge diet (here after called the SARA group) and the cows receiving control diet (Control group).

For ruminal metabolites, ruminal, faecal, and urinary pH, milk fat, milk fat to protein ratio, and blood parameters, data from the control days and full SARA challenge days (days 7–10) were analysed using a repeated measurements model (PROC MIXED, SAS 9.4, SAS Institute Inc., Cary, NC, USA). In the full model, treatment, block, day, time and the interaction between treatment and day and treatment and time were included as fixed explanatory variables; cow ID as random variable, and day as repeated variable. For blood parameters and milk yield, baseline values (mean of values for control days 1–3) were included as a covariate. Coronary band temperature data from days 1–15 was analysed using a repeated measurements model (PROC MIXED, with day as repeated and type = sp(gau) (day) in SAS 9.4). Feed, block, day, room temperature, skin temperature and rectal temperature, and the interaction between feed and day were included as fixed variables, and cow within block as random variable. Model reduction was performed by stepwise backward elimination, removing variables and interactions not significant at *P* < 0.05. Ruminal acetate, propionate, butyrate, valerate, isovalerate, and lactate concentrations were log transformed before analysis to ensure normal distribution. Ruminal contractions, faecal consistency, weight shifting, and dry matter intake data were not normally distributed and a Kruskall Wallis test on a mean score of days 7–10 from each cow was used to analyse whether the distribution differed between SARA and control groups. *P* values < 0.05 were considered significant. Trends were discussed at *P* values between 0.05 and 0.10.

### Ethical considerations

The experiment was planned and performed in a manner aiming to prevent any unnecessary pain and discomfort in the animals. The experiment was approved for animal ethics by the Danish Animal Experiments Inspectorate (file no. 2012-15-2934-00052) prior to performance.

## Results

### Feed

**Figure 1:**
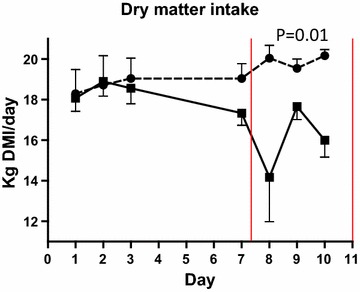
Dry matter intake. Mean dry matter intake (DMI) in cows fed regular TMR diet (*control*, *round* *symbol*, *dot*-and-*dash line*) and TMR + wheat-barley pellets (SARA, *square symbol*, *full line*). SARA cows were fed TMR on days 1–3, TMR + increasing percentages of wheat-barley pellets on days 4–6, full SARA diet (TMR + 40% wheat-barley pellets) on days 7–10 (marked with *red lines*), and TMR on days 11–16. Control cows received TMR diet during the entire period. Differences between groups during full SARA feeding are indicated by P values. *Error bars* SEM.

Dry matter feed intake decreased during the SARA challenge (control: 19.7 kg, SARA: 16.3 kg, P = 0.01) (Figure 1). Feed intake increased after returning to control feeding to reach control levels at day 12. The composition of the TMR used is given in Table 1 and the diet ingredients and chemical composition in Table 2.

### Clinical examination

General demeanour was generally not affected, apart from a few hours of mild to moderate depression in the SARA group, related to the phases of lowest ruminal pH.

**Figure 2:**
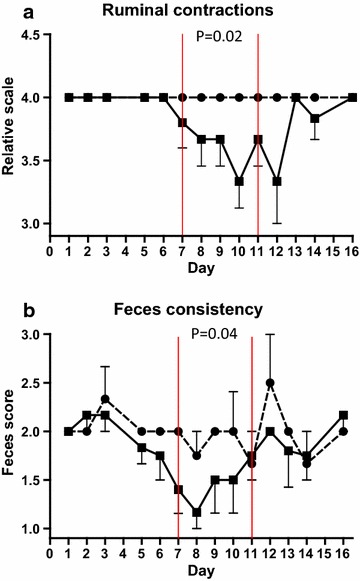
Ruminal contractions and feces consistency. Mean ruminal contractions (**a**) and faecal consistency (**b**) in cows fed regular TMR diet (*control*, *round symbol*, *dot*-and-*dash line*) and TMR + wheat-barley pellets (SARA, *square symbol*, *full line*). SARA cows were fed TMR on days 1–3, TMR + increasing percentages of wheat-barley pellets on days 4–6, full SARA diet (TMR + 40% wheat-barley pellets) on days 7–10 (marked with *red lines*), and TMR on days 11–16. Control cows received TMR diet during the entire period. Differences between groups during full SARA feeding are indicated by P values. *Error bars* SEM.

Ruminal contractions decreased in the SARA group compared to control group from day 6 to day 10 (lowest mean score 3.3) and thereafter increased to reach control levels at day 13 (*P* = 0.02) (Figure 2a). Faecal consistency decreased in the SARA group from day 6 to day 8 (lowest mean score 1.2) and thereafter increased to reach control levels at day 11 (*P* = 0.04) (Figure 2b). Rectal temperature (38.6°C in both groups), heart rate (Control: Mean 78.9 beats per minute, SARA challenge: 80.7) and respiration rate (Mean Control: 32, SARA: 30 respirations per minute) did not differ between SARA and control groups.

### Orthopaedic examination

**Figure 3:**
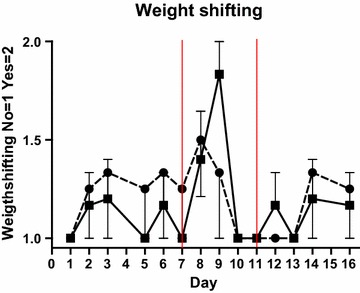
Weight-shifting behaviour. Mean weight-shifting behaviour in cows fed regular TMR diet (*control*, *round symbol*, *dot*-and-*dash line*) and TMR + wheat-barley pellets (SARA, *square symbol*, *full line*). SARA cows were fed TMR on days 1–3, TMR + increasing percentages of wheat-barley pellets on days 4–6, full SARA diet (TMR + 40% wheat-barley pellets) on days 7–10 (marked with *red lines*), and TMR on days 11–16. Control cows received TMR diet during the entire period. Differences between groups during full SARA feeding are indicated by P values. *Error bars* SEM.

Overall, weight-shifting behaviour did not differ between groups, however a numeric increase in cows on SARA challenge shifting weight was observed on day 9 (Figure 3). Coronary band temperature did not differ between groups (control: 29.3°C, SARA challenge: 29.3°C, *P* = 0.97). There was an effect of day (*P* = 0.0006), room temperature (*P* < 0.0001) and skin temperature on the thigh (*P* = 0.005) but not of rectal temperature. None of the cows reacted to claw testing at any point during the trial. All cows were scored non-lame (1) or mildly lame (2) according to Sprecher [18] during the trial and there was no difference between groups.

### Ruminal pH

Mean minimum ruminal pH was 5.4 in the SARA group, and 5.8 in the control group (Table 6). The SARA group spent more time than the control with ruminal pH below 5.8 (SARA 490.7 min/day, control 77.6 min/day) and 5.6 (SARA 294.5 min/day, control 11.0 min/day) during the SARA period. Area under the curve of ruminal pH below 5.8 and 5.6 were increased for the SARA group compared to the control group (Table 6). In the SARA group, four out of six inductions resulted in shorter periods (ranging from 10 min to 3 h and 45 min) of ruminal pH between 5.1 and 5.2, and one period of (10 min) between 5.05 and 5.1. One cow spent 18 h with ruminal pH below 5.2 (minimum pH 4.55). In all cows ruminal pH returned to normal without treatment.

**Table 6:** Results of ruminal pH and ruminal fluid metabolites

	Control diet	SARA diet	SED	P values
Ruminal pH				
Mean	6.31	6.06	0.07	0.01
Minimum	5.79	5.36	0.07	0.001
Maximum	6.73	6.77	0.10	0.77
Minutes < pH 5.8	77.6	490.7	112.9	0.01
Minutes < pH 5.6	11.0	294.5	93.4	0.02
Area under the curve < pH 5.8	11.3	143.6	34.0	0.02
Area under the curve < pH 5.6	0	66.5	24.6	0.04
Volatile fatty acids (VFA)				
Total (mM)	129.6	123.6	10.1	0.73
Acetate (mM)	81.9	69.0	6.4	0.02
Propionate (mM)	26.7	30.4	4.4	0.50
Butyrate (mM)	16.3	19.5	1.7	0.25
Valerate (mM)	1.89	2.27	0.44	0.42
Isovalerate (mM)	1.79	1.72	0.20	0.62
Isobutyrate (mM)	1.04	0.76	0.06	<.0001
Caproate (mM)	0.60	0.45	0.16	0.37
l-Lactate (mM)	0.19	0.28	0.15	0.57
Acetate:propionate	3.23	2.52	0.41	0.09

*SED* standard error of difference.

Ruminal pH parameters and concentration of ruminal fluid metabolites from cows fed regular TMR diet (control) and TMR + wheat-barley pellets (SARA). Estimates are given as least squares means from the repeated measurements model. Acetate, propionate, butyrate, valerate, isovalarete, and lactate concentrations were transformed back to the original scale.

### Ruminal metabolites

**Figure 4:**
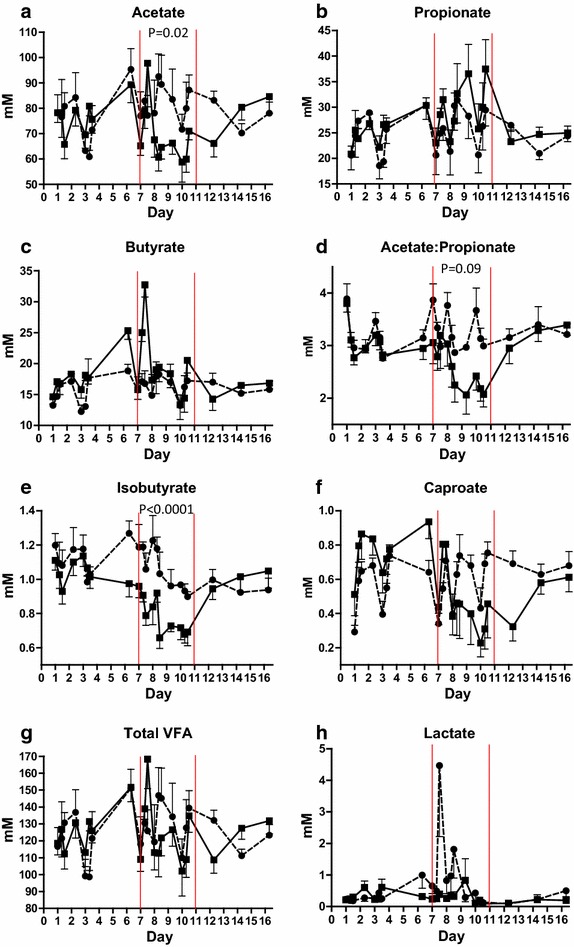
Ruminal fluid VFA (volatile fatty acids) and lactate. Mean concentration of ruminal fluid acetate (**a**), propionate (**b**), butyrate (**c**), acetate to propionate ratio (**d**), isobutyrate (**e**), caproate (**f**), total VFA (**g**) and l-lactate (**h**) from cows fed regular TMR diet (*control*, *round symbol*, *dot*-and-*dash line*) and TMR + wheat-barley pellets (SARA, *square symbol*, *full line*). SARA cows were fed TMR on days 1–3, TMR + increasing percentages of wheat-barley pellets on days 4–6, full SARA diet (TMR + 40% wheat-barley pellets) on days 7–10 (marked with *red lines*), and TMR on days 11–16. Control cows received TMR diet during the entire period. Differences between groups during full SARA feeding are indicated by P values. *Error bars* SEM.

Ruminal fluid acetate concentration decreased from day 8 to day 10 in the SARA group compared to the control group (*P* = 0.02) (Figure 4a; Table 6), but propionate and butyrate concentrations did not differ between SARA and control groups (Figure 4b, c; Table 6). Acetate to propionate ratio tended to be lower (*P* = 0.09) from day 8 to day 10 in the SARA group compared to control (Figure 4d; Table 6). Ruminal isobutyrate concentration significantly decreased (*P* < 0.0001) from day 6 to 10 in the SARA group in comparison to the control group (Figure 4e; Table 6). Caproate concentration was numerically lower in the SARA group compared to control from day 8 to day 12 (Figure 4f; Table 6) and there was an interaction between treatment (SARA challenge) and day (*P* < 0.0001). Total VFA (Figure 4g), valerate, and isovalerate concentrations did not differ between the SARA and control groups (Table 6). Time of the day had a significant effect on acetate, propionate, butyrate, isobutyrate, valerate, caproate, total VFA, and acetate to propionate ratio (*P* < 0.03). Day had a significant effect on butyrate, isobutyrate, valerate, caproate, lactate and acetate to propionate ratio (*P* < 0.05).

Concentrations of l-lactate were generally low (<1.0 mM) and there were no associations with the treatment (SARA challenge) (Table 6). However, in 20% of samples from the SARA group (primarily two cows) l-lactate increased to between 1 and 4 mM, and one sample had a lactate concentration of 14 mM. The highest levels of ruminal l-lactate concentrations were obtained on days 7 and 8 at 15.00 and 21.00 (Figure 4h). There was an interaction between diet and time of the day (*P* = 0.04) on the concentration of lactate in ruminal fluid.

### Faecal and urinary pH

**Figure 5:**
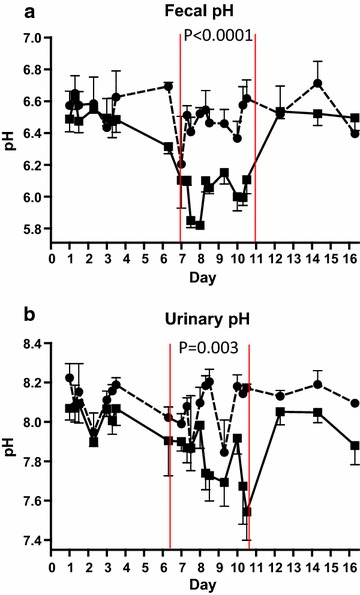
Faecal and urinary pH. Mean faecal pH (**a**) and urinary pH (**b**) in cows fed regular TMR diet (*control*, *round symbol*, *dot*-and-*dash line*) and TMR + wheat-barley pellets (SARA, *square symbol*, *full line*). SARA cows were fed TMR on days 1–3, TMR + increasing percentages of wheat-barley pellets on days 4-6, full SARA diet (TMR + 40% wheat-barley pellets) on days 7–10 (marked with *red lines*), and TMR on days 11–16. Control cows received TMR diet during the entire period. Differences between groups during full SARA feeding are indicated by P values. *Error bars* SEM.

Faecal pH was decreased in the SARA group (pH 6.04) compared to the control group (pH 6.49) (*P* < 0.0001). There was an overall effect of time of the day on faecal pH (*P* = 0.03) although the direction of the effect was not obvious (Figure 5a; Table 7).

**Table 7:** Results of faecal, urinary and blood parameters

	Control diet	SARA diet	SED	P values
Faecal pH	6.49	6.04	0.09	<0.0001
Urinary pH	8.05	7.80	0.19	0.003
Blood				
PCV (%)	27.5	29.2	0.7	0.02
Plasma protein (g/L)	72.0	75.4	2.4	0.18
pCO_2_ (mmHg)	46.5	48.3	1.1	0.10
pO_2_ (mmHg)	41.8	39.6	4.1	0.26
pH	7.370	7.367	0.011	0.73
Ionized calcium (mM)	1.30	1.25	0.01	0.0005
Potassium (mM)	3.79	3.70	0.22	0.55
Sodium (mM)	140.6	140.4	na	0.89
Chloride (mM)	102.9	102.0	1.6	0.23
Glucose (mM)	3.83	3.93	na	0.99
Lactate (mM)	0.61	0.65	na	0.78

*SED* standard error of difference, *na* not available as non parametric test was used.

Faecal, urinary and blood parameters from cows fed regular TMR diet (control) and TMR + wheat-barley pellets (SARA). Estimates are given as least squares means from the repeated measurements model (for sodium, glucose and lactate arithmetic mean and P-values from Kruskall Wallis test are given).

Urinary pH was decreased in the SARA group (pH 7.80) compared to control group (pH 8.05) (*P* = 0.003). There was a significant interaction between treatment and day (*P* = 0.04) seen as progressively lower urinary pH from day 7 (pH 7.93) to day 10 (pH 7.71) in the SARA group (Figure 5b; Table 7). The interaction between treatment and time of day tended to be significant (*P* = 0.07) indicating that progressively lower urinary pH from 9.00 h (pH 7.95) to 21.00 h (pH 7.71) occurred in the SARA group.

### Blood parameters

**Figure 6:**
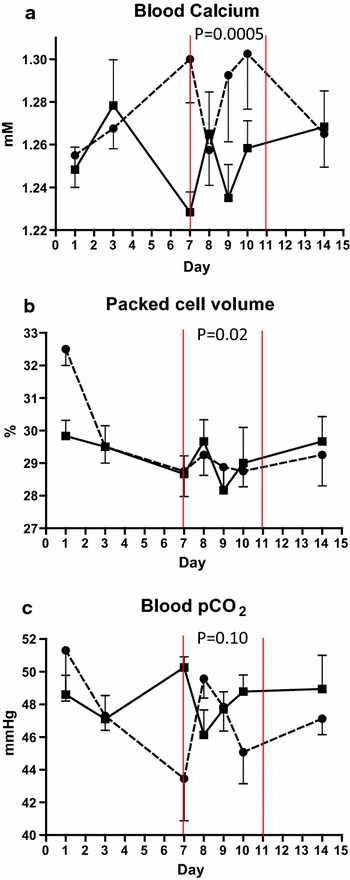
Blood calcium, packed cell volume and pCO_2_. Mean blood ionized calcium concentration (**a**), packed cell volume (PCV) (**b**), and pCO_2_ (**c**) in cows fed regular TMR diet (*control*, *round symbol*, *dot*-and-*dash line*) and TMR + wheat-barley pellets (SARA, *square symbol*, *full line*). SARA cows were fed TMR on days 1–3, TMR + increasing percentages of wheat-barley pellets on days 4–6, full SARA diet (TMR + 40% wheat-barley pellets) on days 7–10 (marked with *red lines*), and TMR on days 11–16. Control cows received TMR diet during the entire period. Differences between groups during full SARA feeding are indicated by P values. *Error bars* SEM.

Blood ionized calcium concentrations were lower in samples from the SARA group (1.25 mM) compared to control group (1.30 mM) (P = 0.0005, Figure 6a; Table 7).

PCV in samples from the SARA group was higher (29.2%) than the control group (27.5%) (*P* = 0.02) (Figure 6b; Table 7).

Overall, blood pH did not differ between SARA and control groups (Table 7), however there was a slight decrease in blood pH from day 7 (7.387) to day 10 (7.349) in the SARA group and the interaction between treatment and day tended to be significant (*P* = 0.10).

Mean blood pCO_2_ was 46.5 mmHg in the control group and 48.3 mmHg in the SARA group however this difference was not significant (*P* = 0.10). There was an interaction between treatment and day (*P* = 0.008) but numerically the direction of the effect was not obvious (Figure 6c; Table 7).

Concentrations of potassium, sodium, chloride, glucose, lactate, blood pO_2_, or plasma protein did not differ between groups (Table 7).

### Milk yield and components

**Figure 7:**
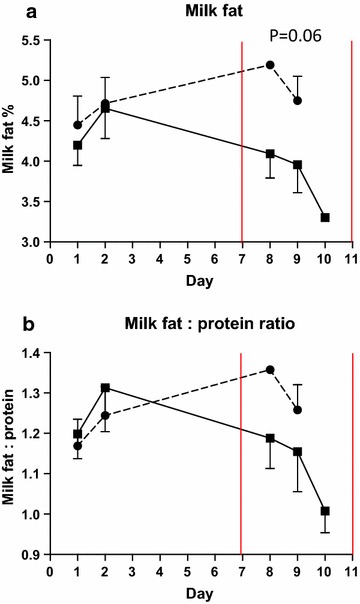
Milk fat content and milk fat to protein ratio. Mean milk fat content (**a**), and milk fat to milk protein ratio (**b**) in cows fed regular TMR diet (*control*, *round symbol*, *dot*-and-*dash line*) and TMR + wheat-barley pellets (SARA, *square symbol*, *full line*). SARA cows were fed TMR on days 1–3, TMR + increasing percentages of wheat-barley pellets on days 4–6, full SARA diet (TMR + 40% wheat-barley pellets) on days 7–10 (marked with *red lines*), and TMR on days 11–16. Control cows received TMR diet during the entire period. Differences between groups during full SARA feeding are indicated by P values. *Error bars* SEM.

Daily milk yield (control: 22.8 kg, SARA: 20.8 kg, *P* = 0.23) did not differ between groups. Milk fat content was decreased in the SARA group (4.14%) compared to the control group (5.08%, *P* = 0.06) (Figure 7a). Milk fat to milk protein ratio decreased numerically during the SARA challenge (control: 1.37, SARA: 1.21, P = 0.24) and there was an interaction between the SARA challenge and time of the day (*P* = 0.04) on this ratio (Figure 7b). Milk protein content did not differ between groups (control 3.65%, SARA: 3.45%, *P* = 0.31).

## Discussion

### Induction of SARA

The challenge used to induce SARA was based on the grain-based SARA challenges used by Gozho et al. [6], Khafipour et al. [7], and Li et al. [14]. The objective was to create a reversible ruminal pH depression below 5.6 for more than 180 min/day, preferably without a drop in ruminal pH below 5.2. These thresholds were set, as ruminal pH depressions below 5.6 for less than 180 min/day are not associated with increase in acute phase proteins in blood and bacterial endotoxin in ruminal fluid, and ruminal pH below 5.2 may indicate acute ruminal acidosis [4, 6]. During the SARA challenge, the ruminal pH was below 5.6 for an average of 294.5 min/day (Table 6). Four cows experienced shorter periods of ruminal pH just below the 5.2 threshold and one cow spent a longer period below the desired pH. Ruminal pH returned to control levels after return to control feeding without intervention in all cases. Hence, based on the ruminal pH depression, we succeeded in inducing SARA, with shorter bouts of acute ruminal acidosis in four cows and one longer period of acute acidosis in one cow. These results underline the large individual variation in the ability of cows to deal with the same level of high grain feeding. The SARA challenge was also accompanied with reductions in feed intake and milk fat content, which are common signs of SARA [3, 4, 14]. Lactate concentrations in ruminal content remained below 5 mM with the exception of one sample, which we considered an outlier. Reversible decreases in ruminal contractions, faecal consistency, ruminal acetate and acetate to propionate ratio, milk fat content, and milk fat to milk protein ratio were also observed, all of which have been associated with SARA [3, 4, 14]. Hence, according to these criteria we conclude that our SARA challenge induced SARA on a relevant Danish production diet background.

### Effects of SARA challenge on clinical parameters and measures in faeces, urine, and blood

In the current study only a small number of animals were included due to the high cost and difficult logistics of dealing with surgically modified animals. This limits the statistical power and, thereby, the likelihood that small, actual differences between groups will be detected and found significant; and subsequently restricts our ability to definitively conclude from the results when no difference between groups was found. However, as mentioned earlier, we believe that only measures that are greatly affected by SARA, i.e. results in large differences between SARA and control groups, offer perspective as diagnostic markers for this disorder. In the current study, the parameters that did not come out significant were all far from significance.

The SARA challenge was not associated with lameness, weight shifting, reaction to hoof testing, or increased coronary band temperature in this study. Changes in these parameters would have been most likely to represent signs of acute inflammation (pain and increased temperature) in the dermal components of the foot. Data from the same animals on solar and white line haemorrhages and elasticity and puncture resistance of claw horn samples from 4 to 10 weeks after SARA feeding was analysed in another study and no difference between groups could be detected [20]. Acute ruminal acidosis has previously been associated with increase in locomotion score and claw pain [21] although subsequent increase in solar and white line haemorrhages was not observed in these animals (Danscher, unpublished results). High grain feeding has also been associated with horn related claw lesions and lameness [11, 12], but even though clinical observations indicate that this association exists, strong evidence from controlled clinical trials is lacking. The absence of changes in orthopaedic relevant parameters in this trial may be due to the short duration of the SARA challenge, the week or non-existing association between low ruminal pH, lameness and claw horn disease, or because these associations are influenced by other factors which were not present in this experimental trial such as physical and environmental stress, and/or hormonal changes around calving.

In this study the cows that received SARA diet had a lower faecal pH than the control group. This is in agreement with Morgante et al. [22] who observed lower faecal pH in herds with an average ruminal pH below 5.8, but contradicts results by Li et al. [14] that showed no effect of SARA induction on faecal pH, and Enemark et al. [15] who concluded that faecal pH was a poor predictor of ruminal pH. High levels of grain feeding may result in carbohydrates including starch bypassing the rumen and reaching the intestines, with subsequent increased fermentation and VFA production in the hindgut causing decreased faecal pH. Information on the level of starch fed in the studies mentioned above was not given. Faecal consistency changed towards more loose/liquid during SARA feeding, but severe diarrhoea was not observed and none of the cows showed clinical signs of dehydration or haemoconcentration.

In this study cows on SARA diet had lower urine pH than control cows. Also, in SARA-challenged cows, urine pH decreased from the first to the last day of the SARA challenge, and urine pH also tended to decrease from morning to evening sampling. Biologically, this can be explained as a response to decreased dietary cation–anion difference (DCAD) in the SARA diet compared to the control diet or to increased acid load on the blood bicarbonate buffer system both resulting increased acid secretion by the kidneys. In herd studies, Gianesella et al. [23] also observed lower pH in cows with ruminal pH below 5.5, but Morgante et al. [22] found no difference in urinary pH between herds with average ruminal pH above and below 5.8, and Enemark et al. [15] concluded that urinary pH was not suitable for predicting low ruminal pH due to the lack of a consistent relationship between the two. In the experimental study by Li et al. [14], when SARA was induced by feeding pellets of ground alfalfa, surprisingly, urinary pH was increased.

Even though there was an association between SARA challenge and decreased fecal and urinary pH in this study, the decreases were small and it is doubtful whether these decreases were large enough to be of diagnostic value. The conflicting results from previous studies [14, 22, 23] emphasize the doubtful value of these parameters as indicators of the SARA syndrome a under field conditions. More research is needed in this area, but it can be argued that in feeding situations where TMR and concentrate are fed separately, and the DCAD value differs between TMR and concentrate, then urinary pH might be a good marker for the ability of cows to balance intake. However, when the DCAD in TMR and concentrate do not differ, urinary pH is probably of little use as a diagnostic measure of the SARA syndrome.

In this study, ionized blood calcium concentration was decreased in SARA-challenged cows compared to control cows. The change was, however, small (1.25 vs. 1.30 mM) and values remained within the reference range (1.2–1.6 mM [24]). Hypocalcaemia is known to be associated to endotoxaemia [25] and decreased blood pH, both conditions which may be present in SARA. Other studies found no difference in blood calcium between SARA and control groups [14, 22].

Numerically there was no difference in PCV values between diet groups during the time of the SARA challenge (Figure 6b). Samples from SARA challenged cows had higher PCV values than control cows in the control period, resulting in an apparent (and statistical), but not actual, effect of treatment group. The differences were small, and all values were within standard reference values (24–46% [24]). The response is in concordance with earlier studies that showed no significant effect of SARA on PCV [14, 23, 26, 27] and Goad [28] who observed a slight decrease in PCV 36–48 h after inducing SARA in hay-adapted steers.

In the current study, there was no difference in blood pH between the two groups. The slight decrease observed from day 7 to day 10 in SARA-challenged cows, might indicate an increase in acid load that was partly buffered. The change was small (7.39–7.35) and within reference ranges (7.35–7.50 [24]). These results are in agreement with earlier studies which showed no or only minimal effect of SARA on blood pH [14, 26, 28].

In the current study, blood pCO_2_, tended to be higher in the SARA group compared to the control group. This can biologically be explained as a response to increased acid load on the bicarbonate buffer system. Most pCO_2_ values for both SARA-challenged and control cows were above the reference range (34–45 mmHg [24]). The interaction between treatment and day was significant, but there was no obvious numerical trend for the direction of the effect. This might be explained by a significant effect of the baseline (control) value covariate on pCO_2_. Increases in blood CO_2_ in SARA-challenged cows have also been described by Li et al. [14]. Herds with average ruminal pH below 5.8 had higher pCO_2_ values than in herds with pH above 5.8 [22] and cows with ruminal pH below 5.5 had higher pCO_2_ values than cows with pH above 5.8 [23]. Brown et al. [26] found no difference in pCO_2_ values in steers induced with SARA compared to controls.

### Markers of SARA

The association between SARA challenge and a tendency to increased pCO_2_ is in agreement with previous studies, whereas the literature on the effect of SARA on urinary and faecal pH, PCV and calcium are more varied. These measures may be helpful to diagnose the SARA syndrome when serial measurements are conducted. However, careful use of the results is warranted, as the parameters do not seem to change consistently, changes were small even though some cows in this study experienced periods of pH below ruminal pH 5.2, and most parameters remained within normal ranges. The biological significance of these results needs to be investigated further. Physiological diurnal fluctuations in faecal and urinary pH, reflecting variations in feed intake, transit time, fermentation patterns, absorption of metabolites and pH in rumen and hindgut during the day, further complicates the use of daily spot samples as indicators of SARA, or at least of a ruminal pH depression, for practical herd use.

Our relatively inconclusive results probably reflect that the SARA syndrome is not a well-defined disease and no specific pathognomonic signs have been identified. No generally agreed diagnostic measure of SARA exists. So far the most concrete suggestion for a definition of SARA has been a ruminal content pH depression below a certain threshold for a certain length of time, for instance below 5.6 for >3 h/day [6]. This definition of SARA was developed in a research setting and may be less representative for cases in the field. Additionally, it requires continuous measurement of ruminal pH, which is a practical and economical challenge under field conditions. Additionally, it is not established that low ruminal pH alone is sufficient to induce SARA, or if other factors needs to be present to prompt the development of SARA-related symptoms such as feed intake depression and inflammation. Prediction of SARA-related signs is further complicated by a large individual variation in susceptibility towards low ruminal pH, which may be due to individual differences in genetics, microbiota of the digestive tract, feeding behavior, capacity for absorption of VFA, etc. [4, 29]. Studies have shown that low ruminal pH induced by feeding pellets of ground alfalfa may reduce milk yield and milk fat but in itself does not seem to be enough to induce systemic inflammation and translocation of LPS [13]. Cow foraging solely on pasture have been shown to experience low ruminal pH, altered faecal consistency and low milk fat content [30], yet to our knowledge, SARA-related symptoms is not generally reported as a major problem in pasture based production systems. This can be due to under-diagnosing or it can be speculated whether the higher production level, the predominantly starch-based feeding, or other factors in the TMR based systems interacts with the low ruminal pH in the development of the SARA syndrome, or if hind gut acidosis may play a role [29]. Nevertheless, it indicates that depression of the ruminal pH is only one of several factors involved in development of SARA-related symptoms. SARA is thus an ill-defined concept and may be regarded merely a clinical phenotype related to high grain feeding. There is epidemiological evidence that this phenotype is more prone to other diseases. Probably a continuum exists between the well-functioning rumen and physiology of a healthy, high yielding dairy cow in maximal production and the ecological and physiological breakdown of a cow with full blown acute ruminal and systemic acidosis—with the “high-concentrate syndrome” [31] /SARA condition, situated in between theses extremes. The complexity of such a continuum is probably best described by a combination of known indicators, for example in a risk index. The main challenge in the development of such an index remains, however, the lack of a “gold standard” for the diagnosis of the SARA syndrome.

## Conclusions

In the current study, the SARA challenge, defined by a diet capable of reducing ruminal pH, could not be related to lameness or acute signs of claw inflammation or claw pain. The challenge was associated with decreased faecal and urinary pH, increased blood calcium and PCV and a tendency to increased blood pCO_2_. These measures may be helpful for the diagnosis of the SARA syndrome when serial measurements are conducted. However, careful use of the results is warranted, as the parameters do not seem to change consistently, changes were small, and diurnal variations were present in faecal and urinary pH. Due to the small number of cows in the study, the statistical power to find small differences between groups as significant was limited. However, for a measure to act as an accurate diagnostic tool, these differences must be substantial. None of these parameters seem to be able to stand alone as indicators of the SARA syndrome. However, measures could be combined with other indicators to a combined risk index for compromised gut health. Large field trials are needed before these parameters, or a combined risk index, can be recommended for the diagnosis of the SARA syndrome on dairy farms.
